# Palbociclib in combination with letrozole in patients with estrogen receptor–positive, human epidermal growth factor receptor 2–negative advanced breast cancer: PALOMA-2 subgroup analysis of Japanese patients

**DOI:** 10.1007/s10147-018-1353-9

**Published:** 2018-12-04

**Authors:** Hirofumi Mukai, Chikako Shimizu, Norikazu Masuda, Shoichiro Ohtani, Shinji Ohno, Masato Takahashi, Yutaka Yamamoto, Reiki Nishimura, Nobuaki Sato, Shozo Ohsumi, Hiroji Iwata, Yuko Mori, Satoshi Hashigaki, Yasuaki Muramatsu, Takashi Nagasawa, Yoshiko Umeyama, Dongrui R. Lu, Masakazu Toi

**Affiliations:** 1 Division of Breast and Medical Oncology, National Cancer Center Hospital East, 6-5-1 Kashiwanoha, Kashiwa-shi, Chiba, 277-8577 Japan; 2 National Cancer Center Hospital, Tokyo, Japan; 3 National Hospital Organization Osaka National Hospital, Osaka, Japan; 4 Hiroshima City Hiroshima Citizens Hospital, Hiroshima, Japan; 5 The Cancer Institute Hospital of JFCR, Tokyo, Japan; 6 National Hospital Organization Hokkaido Cancer Center, Hokkaido, Japan; 7 Kumamoto University Graduate School of Medical Sciences, Kumamoto, Japan; 8 Kumamoto Shinto General Hospital, Kumamoto, Japan; 9 Niigata Cancer Center Hospital, Niigata, Japan; 10 National Hospital Organization Shikoku Cancer Center, Ehime, Japan; 11 Aichi Cancer Center Hospital, Aichi, Japan; 12 Pfizer Japan Inc, Tokyo, Japan; 13 Pfizer Oncology, San Diego, CA USA; 14 Kyoto University Graduate School of Medicine, Kyoto, Japan

**Keywords:** Advanced breast cancer, HER2−, HR+, Japanese, Letrozole, Palbociclib

## Abstract

**Background:**

In PALOMA-2, palbociclib–letrozole significantly improved progression-free survival (PFS) vs placebo–letrozole in women with estrogen receptor–positive, human epidermal growth factor receptor 2–negative (ER+/HER2–) advanced breast cancer (ABC) in the first-line setting. We evaluated the efficacy, safety, and pharmacokinetics of palbociclib in Japanese women in PALOMA-2.

**Methods:**

In this phase 3 study, 666 postmenopausal women with ER+/HER2– ABC were randomized 2:1 to palbociclib (125 mg/day [3 weeks on/1 week off]) plus letrozole (2.5 mg daily) or placebo plus letrozole. A prespecified, exploratory, subgroup analysis of Japanese patients (*n* = 46) was conducted to compare results with those of the overall population.

**Results:**

At the February 26, 2016 cutoff, median PFS among the 46 Japanese patients was 22.2 months (95%CI, 13.6‒not estimable) with palbociclib–letrozole vs 13.8 months (5.6‒22.2) with placebo–letrozole (hazard ratio, 0.59 [95%CI, 0.26−1.34]). The most common adverse events (AEs) were hematologic and more frequent among Japanese patients than the overall population (neutropenia: 93.8% [87.5% grade 3/4] vs 79.5% [66.4%]; leukopenia: 62.5% [43.8%] vs 39.0% [24.8%]); no Japanese patients had febrile neutropenia. Palbociclib dose reductions due to toxicity (mainly neutropenia) were more common in Japanese patients (62.5% vs 36.0%); few permanently discontinued due to AEs. Although mean palbociclib trough concentration was higher in Japanese patients vs non-Asians (95.4 vs 61.7 ng/mL), the range of individual values of the Japanese patients was within that of non-Asians.

**Conclusions:**

These results from PALOMA-2 suggest that palbociclib–letrozole merits consideration as a first-line treatment option for postmenopausal Japanese patients with ER+/HER2‒ ABC. ClinicalTrials.gov: NCT01740427.

**Electronic supplementary material:**

The online version of this article (10.1007/s10147-018-1353-9) contains supplementary material, which is available to authorized users.

## Introduction

Breast cancer is the most common cancer and fifth leading cause of cancer-related death in Japan [1]. Hormone receptor‒positive (HR+) breast cancer accounts for approximately 71% of new cases worldwide [2]. Endocrine therapy is a mainstay of HR+ breast cancer treatment and is recommended by the Japanese Breast Cancer Society and international guidelines for initial treatment of HR+ disease [3, 4]. However, hormonal blockade alone provides only modest benefit in women with advanced breast cancer (ABC), with many patients having de novo or acquired resistance to endocrine therapy [4].

Palbociclib is a selective, oral inhibitor of cyclin-dependent kinases 4 and 6 (CDK4/6) that prevents cell proliferation by blocking cell cycle progression from the G1 to the S phase [5]. In the multinational, phase 3 PALOMA-2 study, median progression-free survival (PFS) was significantly longer with palbociclib–letrozole than with placebo–letrozole as first-line treatment for postmenopausal women with estrogen receptor−positive (ER+)/human epidermal growth factor receptor 2‒negative (HER2‒) ABC, and toxicities were manageable [6]. Palbociclib is approved in the United States, the European Union, and Japan for the treatment of HR+/HER2‒ ABC in combination with endocrine therapy [7–9].

Limited data are available on the efficacy and safety of palbociclib–letrozole in Japanese patients. A phase 1 study in postmenopausal Japanese women with ER+/HER2– ABC determined that the optimal dosage of palbociclib is 125 mg once daily (QD) for 3 weeks on followed by 1 week off (3/1 schedule), the same as in Western patients [10]. A single-arm, phase 2 study evaluated the efficacy and safety of palbociclib–letrozole (3/1 regimen) in 42 postmenopausal Japanese patients with treatment-naive ER+/HER2‒ ABC; although follow-up is ongoing, initial results are encouraging with a 1-year probability of PFS of 75.0% (90%CI, 61.3%−84.4%) [11].

Data on the efficacy and safety of palbociclib–letrozole in Asian patients in PALOMA-2 have been reported [12]; however, some patients resided in Western countries, as the analysis included all patients who reported Asian ethnicity. Dietary habits and medical environments vary among Asian countries, and these factors may influence clinical outcomes [13, 14]. Therefore, additional investigation in Japanese patients is warranted. This paper presents results of a prespecified exploratory analysis of the efficacy—including the association between efficacy and dose reduction—safety, and pharmacokinetics (PK) of palbociclib in Japanese women residing in Japan enrolled in PALOMA-2, as well as ad hoc analyses in the overall population of factors associated with neutropenia, the most common adverse event (AE) associated with palbociclib.

## Patients and methods

### Study design and patients

The PALOMA-2 study has been described previously [6]. Briefly, postmenopausal women with histologically or cytologically confirmed ER+/HER2‒ ABC (locoregionally recurrent or metastatic) not suitable for resection or radiation therapy with curative intent were randomized 2:1 to receive palbociclib 125 mg QD (3/1 schedule) plus daily letrozole 2.5 mg or matching placebo plus letrozole. Dose reductions of palbociclib or placebo due to AEs were allowed; dose reductions of letrozole were not. Randomization was stratified by disease site (visceral/nonvisceral), disease-free interval from the end of (neo)adjuvant therapy (de novo metastatic, ≤ 12 months, > 12 months), and prior (neo)adjuvant hormonal therapy (yes, no). There was no stratification by country/region.

Patients included in this analysis were residing in Japan. The study was approved by an institutional review board or ethics committee at each site (Table S1), and all patients provided written informed consent. PALOMA-2 was conducted in accordance with the Declaration of Helsinki and the International Conference on Harmonisation Good Clinical Practice Guidelines.

### Outcomes and assessments

The primary endpoint was investigator-assessed PFS. Secondary endpoints included objective response (confirmed partial or complete response per RECIST v1.1), clinical benefit response (CBR; objective response or stable disease for ≥ 24 weeks per RECIST v1.1), safety, and PK. Radiological tumor assessments were performed at screening and every 12 weeks during treatment.

Laboratory analyses were performed on days 1 and 15 of cycles 1 and 2 then on day 1 of each subsequent cycle. AEs were assessed according to the National Cancer Institute Common Terminology Criteria for Adverse Events, version 4.0. Blood samples for PK analysis were obtained predose on day 15 of cycles 1 and 2. Plasma samples were analyzed using a validated high-performance liquid chromatography with tandem mass spectrometry.

### Statistical analyses

Estimates of median PFS and corresponding 2-sided 95% confidence intervals were obtained using the Kaplan–Meier method. Cox proportional hazard models were used to calculate HRs [6]. Comparisons of PFS were done using 1-sided unstratified log-rank tests. A blinded independent central review (BICR) of PFS was conducted for all patients as a supportive analysis. Safety data are summarized using descriptive statistics in patients who received ≥ 1 dose of study treatment (as-treated population). Nominal *P* values are presented, and no adjustments were made for multiple testing. Additional details are published elsewhere [6]. Pearson correlation coefficients were calculated for the analyses evaluating the relationship between palbociclib trough concentration (*C*_trough_) and body weight/body surface area (BSA)/body mass index (BMI) and factors associated with posttreatment neutrophil counts.

## Results

### Patients and study treatment

Between December 2013 and June 2014, 46 Japanese patients enrolled in PALOMA-2; 32 were randomized to palbociclib–letrozole and 14 to placebo–letrozole. Demographics and baseline disease characteristics of the overall and Japanese populations are in Table 1. Japanese women had a lower median body weight compared with the overall population (palbociclib–letrozole, 53.9 vs 68.0 kg; placebo–letrozole, 57.1 vs 66.8 kg) and a better Eastern Cooperative Oncology Group (ECOG) performance status (palbociclib–letrozole, 84.4% vs 57.9% had ECOG performance status grade 0; placebo–letrozole, 71.4% vs 45.9%). More Japanese patients vs the overall population had visceral disease (palbociclib–letrozole, 62.5% vs 48.2%; placebo–letrozole, 71.4% vs 49.5%) and a disease-free interval > 12 months (palbociclib–letrozole, 59.4% vs 40.1%; placebo–letrozole, 64.3% vs 41.9%). In the palbociclib–letrozole group, more Japanese patients (62.5%) were ≥ 65 years of age (vs 40.8% in the overall population).

**Table 1:** Demographics and baseline disease characteristics in the overall population and Japanese patients

Characteristic	Overall population		Japanese patients	
	PAL + LET (*n* = 444)	PBO + LET (*n* = 222)	PAL + LET (*n* = 32)	PBO + LET *(n* = 14)
Age, y				
Median (range)	62 (30‒89)	61 (28‒88)	67 (44‒88)	61 (51‒88)
< 65, *n* (%)	263 (59.2)	141 (63.5)	12 (37.5)	11 (78.6)
≥ 65, *n* (%)	181 (40.8)	81 (36.5)	20 (62.5)	3 (21.4)
Median (range) weight, kg	68.0 (33.0‒156.8)	66.8 (35.0‒124.8)	53.9 (33.0‒88.0)	57.1 (43.8‒67.4)
ECOG performance status, *n* (%)				
0	257 (57.9)	102 (45.9)	27 (84.4)	10 (71.4)
1	178 (40.1)	117 (52.7)	3 (9.4)	4 (28.6)
2	9 (2.0)	3 (1.4)	2 (6.3)	0
Disease site,^a^*n* (%)				
Visceral^b^	214 (48.2)	110 (49.5)	20 (62.5)	10 (71.4)
Nonvisceral	230 (51.8)	112 (50.5)	12 (37.5)	4 (28.6)
Bone-only	103 (23.2)	48 (21.6)	4 (12.5)	1 (7.1)
Number of disease sites, *n* (%)				
1	138 (31.1)	66 (29.7)	7 (21.9)	5 (35.7)
2	117 (26.4)	52 (23.4)	10 (31.3)	4 (28.6)
3	112 (25.2)	61 (27.5)	12 (37.5)	3 (21.4)
≥ 4	77 (17.3)	43 (19.4)	3 (9.4)	2 (14.3)
Disease-free interval,^a,c^*n* (%)				
Newly metastatic disease	167 (37.6)	81 (36.5)	8 (25.0)	3 (21.4)
≤ 12 months	99 (22.3)	48 (21.6)	5 (15.6)	2 (14.3)
> 12 months	178 (40.1)	93 (41.9)	19 (59.4)	9 (64.3)
Prior (neo)adjuvant therapy,^a^*n* (%)				
Hormonal therapy	249 (56.1)	126 (56.8)	21 (65.6)	10 (71.4)
Chemotherapy	213 (48.0)	109 (49.1)	15 (46.9)	8 (57.1)

*ECOG* Eastern Cooperative Oncology Group, *LET* letrozole, *PAL* palbociclib, *PBO* placebo

^a^Based on case report form data

^b^Refers to lung (including pleura) or liver involvement

^c^Calculated as the time between end of neoadjuvant or adjuvant treatment and onset of metastatic disease or disease recurrence

In contrast with the overall population, median duration of treatment among Japanese patients was similar with both treatments (palbociclib–letrozole, 13.34 [range, 0.69–26.15] months; placebo–letrozole, 13.60 [2.04–24.87] months; Table 2). More Japanese patients required palbociclib dose reductions (62.5% vs 36.0% overall), leading to a lower relative dose intensity than the overall population (74.3% [44.0%‒100.0%] vs 93.0% [40.3%–109.5%]).

**Table 2:** Exposure to study drug in the overall population and Japanese patients^a^

	Overall population		Japanese patients	
	PAL + LET (*n* = 444)	PBO + LET (*n* = 222)	PAL + LET (*n* = 32)	PBO + LET (*n* = 14)
PAL or PBO				
Duration of treatment,^b^ median (range), months	19.81 (0.03–34.07)	13.57 (0.33–35.18)	13.34 (0.69–26.15)	13.60 (2.04–24.87)
Average daily dose, median (range), mg	125.0 (76.6–125.2)	125.0 (104.7–125.6)	112.4 (84.4–125.0)	125.0 (109.6–125.6)
Dose reductions,^c^*n* (%)	160 (36.0)	3 (1.4)	20 (62.5)	1 (7.1)
Reduction to 100 mg	97 (21.8)	3 (1.4)	11 (34.4)	1 (7.1)
Reduction to 75 mg	63 (14.2)	0	9 (28.1)	0
Time to first dose reduction,^d^ median (range), days	90 (28–785)	42 (29–198)	63 (29–785)	42^f^
Dose interruption,^e^*n* (%)	297 (66.9)	92 (41.4)	22 (68.8)	6 (42.9)
Relative dose intensity, median (range), %	93.0 (40.3–109.5)	99.6 (56.1–104.5)	74.3 (44.0–100.0)	99.2 (56.1–100.0)
LET				
Dose interruption,^e^*n* (%)	233 (52.5)	97 (43.7)	20 (62.5)	9 (64.3)
Relative dose intensity, median (range), %	99.9 (73.4–100.2)	100.0 (79.0–100.0)	99.8 (77.8–100.0)	99.9 (93.2–100.0)

*LET* letrozole, *PAL* palbociclib, *PBO* placebo

^a^In the as-treated population

^b^Total number of days from first to and including last day of each study treatment

^c^Includes any dose reduction from the initial prescribed dose; does not include dose interruptions. No changes in letrozole dose were allowed

^d^Timed from start date of first occurrence minus first dose date of cycle 1 + 1

^e^Interruptions include missed dose based on the case report form and dose administered = 0 mg

^f^The range is not applicable, because the dose reduction occurred in one patient

### Efficacy

**Fig. 1:**
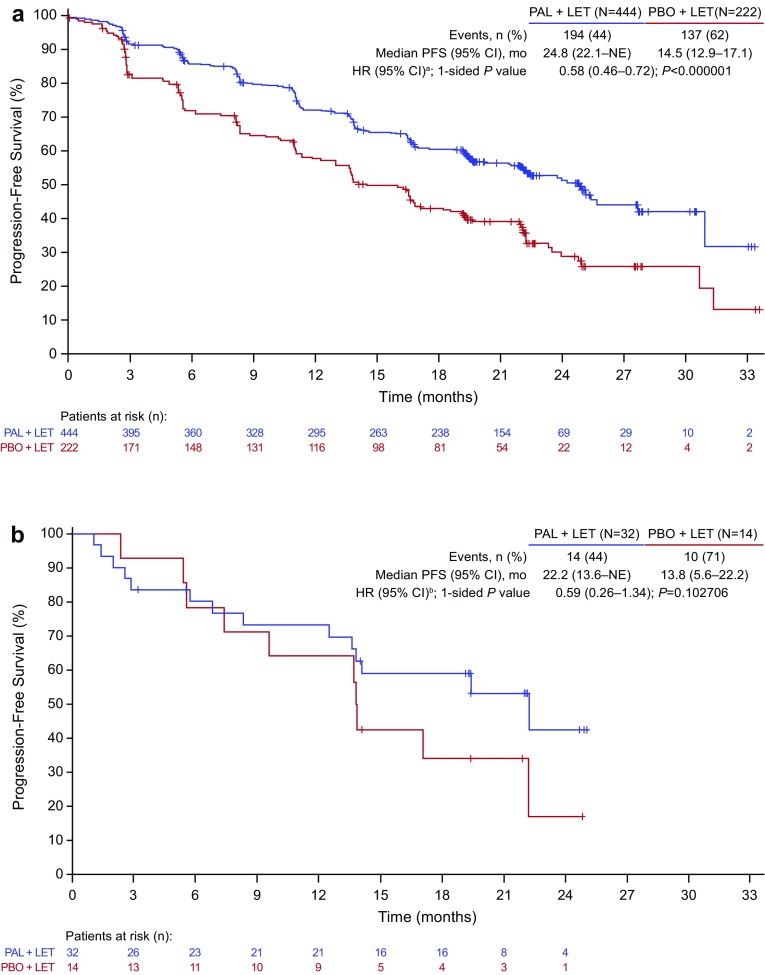
Investigator-assessed PFS in the **a** overall population and **b** Japanese patients (ITT population). *CI* confidence interval, *HR* hazard ratio, *ITT* intent-to-treat, *LET* letrozole, *NE* not estimable, *PAL* palbociclib, *PBO* placebo, *PFS* progression-free survival.** a** HR stratified by disease site (visceral vs nonvisceral) at baseline. **b** Unstratified HR

**Fig. 2:**
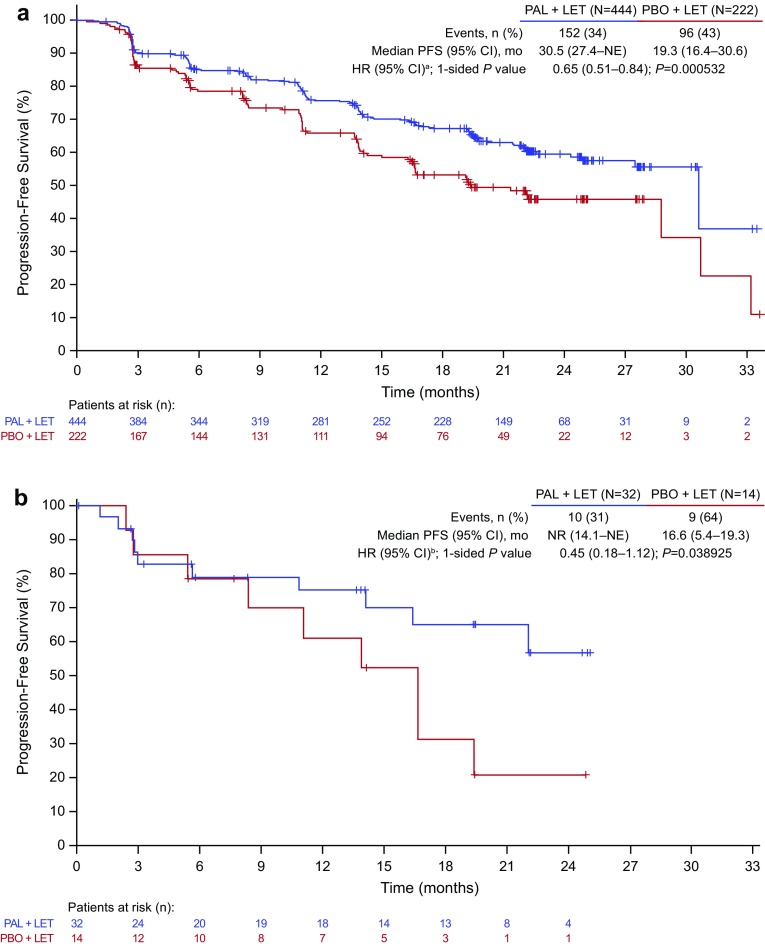
BICR-assessed PFS in the **a** overall population and **b** Japanese patients (ITT population). *BICR* blinded independent central review, *CI* confidence interval, *HR* hazard ratio, *ITT* intent-to-treat, *LET* letrozole, *NE* not estimable, *NR* not reached, *PAL* palbociclib, *PBO* placebo, *PFS* progression-free survival. a HR stratified by disease site (visceral vs nonvisceral) at baseline. **b** Unstratified HR

Median duration of follow-up was similar in the overall (palbociclib–letrozole: 23.0 months; placebo–letrozole: 22.3 months) and Japanese (21.6 and 22.3 months, respectively) populations (data cutoff: February 26, 2016). In the overall population, median PFS was significantly improved with palbociclib–letrozole vs placebo–letrozole (Fig. 1a) [6]. Among Japanese patients, median PFS was 22.2 months (95%CI, 13.6-not estimable) with palbociclib–letrozole vs 13.8 months (5.6–22.2) with placebo–letrozole (HR, 0.59 [95%CI, 0.26–1.34]; 1-sided *P* = 0.103) (Fig. 1b). By BICR, median PFS was 30.5 months (95%CI, 27.4-not estimable) with palbociclib–letrozole vs 19.3 months (16.4–30.6) with placebo–letrozole in the overall population (HR, 0.65 [95%CI, 0.51–0.84]; 1-sided *P* < 0.001; Fig. 2a) [6]. In Japanese patients, BICR median PFS was not reached (95%CI, 14.1 months-not estimable) with palbociclib–letrozole and 16.6 months (5.4–19.3) with placebo–letrozole (HR, 0.45 [95%CI, 0.18–1.12]; 1-sided *P* = 0.039) (Fig. 2b). The PFS with palbociclib was consistent in other Asian patients (excluding Japanese) as well as non-Asians (**Fig. S1**).

Confirmed objective response rate (ORR) was numerically higher with palbociclib–letrozole vs placebo–letrozole in both the overall (55.3% [95%CI, 49.9–60.7] vs 44.4% [36.9–52.2]) and Japanese populations (46.4% [27.5–66.1] vs 38.5% [13.9–68.4]) in patients with measurable disease (Table 3). The degree of improvement in ORR with palbociclib was similar in the intent-to-treat (ITT) population. CBR was higher with palbociclib–letrozole vs placebo–letrozole in the overall population (84.3% [95%CI, 80.0–88.0] vs 70.8% [63.3–77.5]) in patients with measurable disease, but not in Japanese patients (75.0% [95%CI, 55.1–89.3] vs 84.6% [54.6–98.1]); results were similar in the ITT population (Table 3).

**Table 3:** Tumor response in the ITT population and in patients with measurable disease

	Overall population		Japanese patients	
ITT Population	PAL + LET (*n* = 444)	PBO + LET (*n* = 222)	PAL + LET (*n* = 32)	PBO + LET (*n* = 14)
Best overall response, *n* (%)				
Complete response	9 (2.0)	5 (2.3)	1 (3.1)	0
Partial response	178 (40.1)	72 (32.4)	12 (37.5)	5 (35.7)
Stable disease (weeks)	210 (47.3)	96 (43.2)	13 (40.6)	8 (57.1)
≥ 24	190 (42.8)	79 (35.6)	12 (37.5)	7 (50.0)
< 24	20 (4.5)	17 (7.7)	1 (3.1)	1 (7.1)
Disease progression	34 (7.7)	37 (16.7)	5 (15.6)	1 (7.1)
Indeterminate	13 (2.9)	12 (5.4)	1 (3.1)	0
ORR,^a,b^ % (95%CI)	42.1 (37.5–46.9)	34.7 (28.4–41.3)	40.6 (23.7–59.4)	35.7 (12.8–64.9)
Odds ratio^c^ (95%CI)	1.40 (0.98–2.01)		1.23 (0.29–5.79)	
1-sided *P* value	0.0310		0.5095	
CBR,^b,d^ % (95%CI)	84.9 (81.2–88.1)	70.3 (63.8–76.2)	78.1 (60.0–90.7)	85.7 (57.2–98.2)
Odds ratio^c^ (95%CI)	2.39 (1.58–3.59)		0.60 (0.05–3.86)	
1-sided *P* value	< 0.0001		0.8409	

Measurable disease	PAL + LET (*n* = 338)	PBO + LET (*n* = 171)	PAL + LET (*n* = 28)	PBO + LET (*n* = 13)
Best overall response, *n* (%)				
Complete response	9 (2.7)	4 (2.3)	1 (3.6)	0
Partial response	178 (52.7)	72 (42.1)	12 (42.9)	5 (38.5)
Stable disease (weeks)	116 (34.3)	59 (34.5)	9 (32.1)	7 (53.8)
≥ 24	98 (29.0)	45 (26.3)	8 (28.6)	6 (46.2)
< 24	18 (5.3)	14 (8.2)	1 (3.6)	1 (7.7)
Disease progression	25 (7.4)	28 (16.4)	5 (17.9)	1 (7.7)
Indeterminate	10 (3.0)	8 (4.7)	1 (3.6)	0
ORR,^a,b^ % (95%CI)	55.3 (49.9–60.7)	44.4 (36.9–52.2)	46.4 (27.5–66.1)	38.5 (13.9–68.4)
Odds ratio^c^ (95%CI)	1.55 (1.05–2.28)		1.39 (0.30–6.79)	
1-sided *P* value	0.0132		0.4465	
CBR,^b,d^ % (95%CI)	84.3 (80.0–88.0)	70.8 (63.3–77.5)	75.0 (55.1–89.3)	84.6 (54.6–98.1)
Odds ratio^c^ (95%CI)	2.23 (1.39–3.56)		0.55 (0.05–3.63)	
1-sided *P* value	0.0003		0.8650	

*CBR* clinical benefit response, *CI* confidence interval, *ITT* intent-to-treat, *LET* letrozole, *ORR* objective response rate, *PAL* palbociclib, *PBO* placebo

^a^Confirmed complete and partial response

^b^Exact method based on binomial distribution

^c^Stratified and unstratified odds ratio in the overall population and Japanese patients, respectively

^d^Confirmed complete and partial response plus stable disease ≥ 24 weeks

**Fig. 3:**
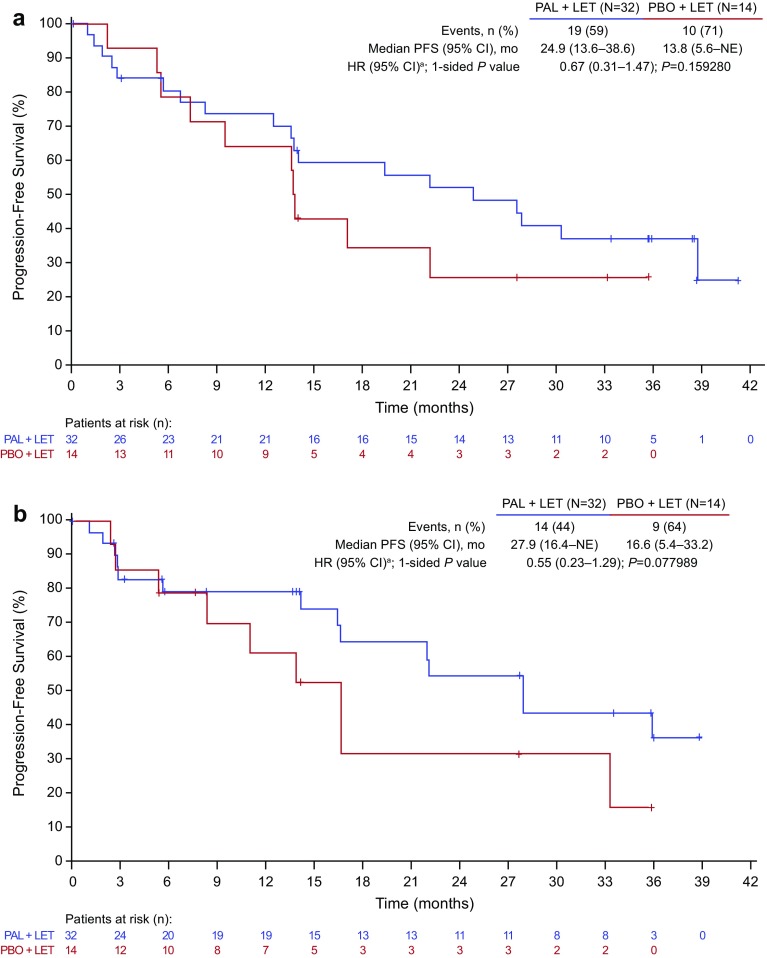
PFS after approximately 37 months median follow-up (data cutoff: May 31, 2017) by **a** investigator assessment and **b** BICR in Japanese patients (ITT population). *BICR* blinded independent central review, *CI* confidence interval, *HR* hazard ratio, *ITT* intent-to-treat, *LET* letrozole, *NE* not estimable, *PAL* palbociclib, *PBO* placebo, *PFS* progression-free survival. ^a^Unstratified HR

Using an updated data cutoff (May 31, 2017), with approximately 37-month median follow-up [15], median investigator-assessed PFS in Japanese patients was 24.9 months (95%CI, 13.6–38.6) with palbociclib–letrozole vs 13.8 months (5.6-not estimable) with placebo–letrozole (HR, 0.67 [95%CI, 0.31–1.47]) (Fig. 3a); median PFS by BICR was 27.9 months (95%CI, 16.4-not estimable) vs 16.6 months (5.4–33.2), respectively (HR, 0.55 [95%CI, 0.23–1.29]) (Fig. 3b). Data for overall survival are not yet mature.

**Fig. 4:**
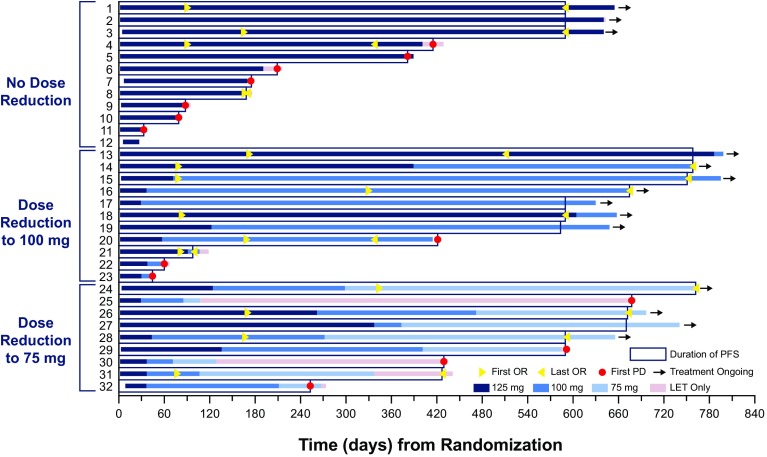
Duration of PFS in 32 Japanese patients treated with palbociclib–letrozole. *OR* objective response, *LET* letrozole, *PD* progressive disease, *PFS* progression-free survival

In the evaluation of the association between efficacy and dose reduction, Japanese patients in the palbociclib–letrozole group who required dose reduction to 100 mg QD or 75 mg QD also showed long PFS (Fig. 4).

### Pharmacokinetics

**Fig. 5:**
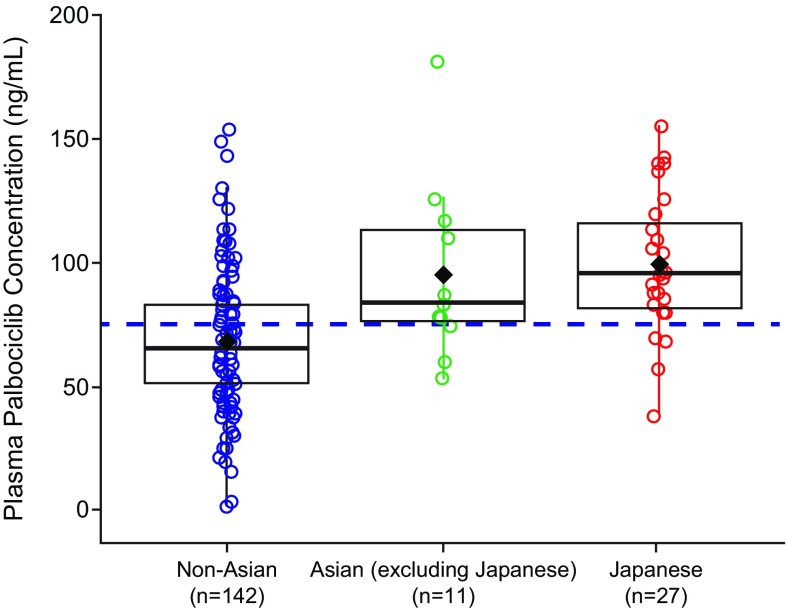
Palbociclib *C*_trough_ at steady state in non-Asian, Asian (excluding Japanese), and Japanese patients. Black diamonds represent the subpopulation arithmetic mean values and open circles represent individual patient values. The dashed blue line represents the arithmetic mean value of all data from all patients. The box plot provides median and 25%/75% quartiles with whiskers to the last point within 1.5 times interquartile range. *C*_*trough*_ trough concentration

**Fig. 6:**
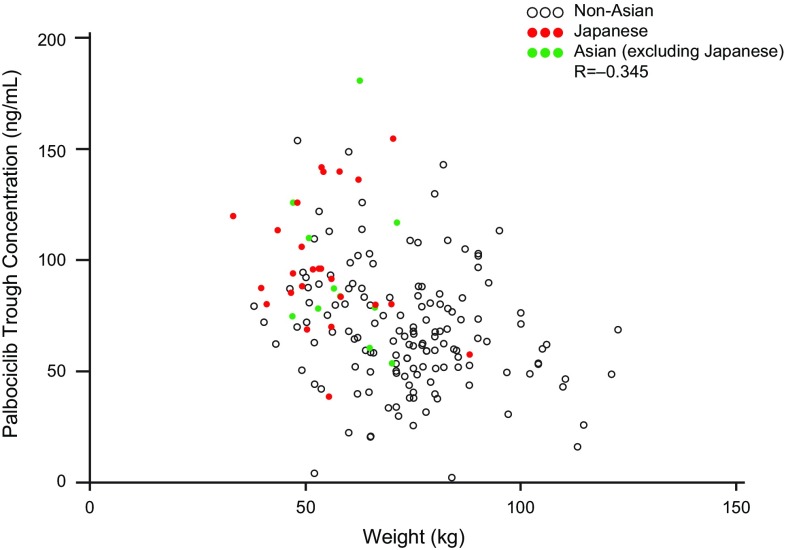
Palbociclib *C*_trough_ at steady state vs body weight in non-Asian, Asian (excluding Japanese), and Japanese patients. Pearson product-moment correlation coefficient (*R*) is presented. Within-patient palbociclib *C*_trough_ are shown. *C*_*trough*_ trough concentration

The geometric mean (geometric CV%) palbociclib *C*_trough_ at steady state was higher in Japanese (95.4 ng/mL [31.3]) and other Asians (90.1 ng/mL [36.0]) relative to non-Asians (61.7 ng/mL [59.1]), indicating greater palbociclib exposure. However, individual palbociclib *C*_trough_ values in each of the 3 groups were generally within a similar range (Fig. 5). No apparent correlation was observed between steady state *C*_trough_ and body weight (Fig. 6) or BSA/BMI (data not shown) in Japanese, Asian (excluding Japanese), and non-Asian patients.

### Safety

Hematologic AEs were the most common toxicities reported with palbociclib–letrozole in the overall and Japanese populations (Table 4). With the exception of neutropenia and leukopenia, most hematologic AEs were of grade 1 or 2 severity. The incidence of any-grade hematologic AEs with combination therapy was higher in the Japanese than in the overall population (neutropenia, 93.8% vs 79.5%; leukopenia, 62.5% vs 39.0%; and thrombocytopenia, 37.5% vs 15.5%, respectively). Grade 3/4 events were more common with palbociclib–letrozole among Japanese patients vs the overall population (neutropenia, 87.5% vs 66.4%; leukopenia, 43.8% vs 24.8%; and thrombocytopenia, 6.3% vs 1.6%). Neutropenia was manageable with dose modifications, and only 3 Japanese women (9.4%) permanently discontinued palbociclib because of this AE. No febrile neutropenia was observed among Japanese patients.

**Table 4:** Adverse events occurring in ≥ 15% of Japanese patients in either arm (all-causality; as-treated population)

Adverse Event	Overall population						Japanese patients					
	PAL + LET (*n* = 444)			PBO + LET (*n* = 222)^b^			PAL + LET (*n* = 32)			PBO + LET (*n* = 14)		
	Any Grade	Grade 3	Grade 4	Any Grade	Grade 3	Grade 4	Any Grade	Grade 3	Grade 4	Any Grade	Grade 3	Grade 4
Any AE, *n* (%)	439 (98.9)	276 (62.2)	60 (13.5)	212 (95.5)	49 (22.1)	5 (2.3)	32 (100)	19 (59.4)	11 (34.4)	13 (92.9)	4 (28.6)	0
Hematologic AEs												
Neutropenia^a^	353 (79.5)	249 (56.1)	46 (10.4)	14 (6.3)	2 (0.9)	1 (0.5)	30 (93.8)	17 (53.1)	11 (34.4)	2 (14.3)	0	0
Leukopenia^a^	173 (39.0)	107 (24.1)	3 (0.7)	5 (2.3)	0	0	20 (62.5)	14 (43.8)	0	1 (7.1)	0	0
Thrombocytopenia^a^	69 (15.5)	6 (1.4)	1 (0.2)	3 (1.4)	0	0	12 (37.5)	2 (6.3)	0	0	0	0
Anemia^a^	107 (24.1)	23 (5.2)	1 (0.2)	20 (9.0)	4 (1.8)	0	9 (28.1)	3 (9.4)	0	3 (21.4)	1 (7.1)	0
Nonhematologic AEs												
Stomatitis^a^	135 (30.4)	4 (0.9)	0	30 (13.5)	0	0	17 (53.1)	0	0	4 (28.6)	0	0
Nasopharyngitis	62 (14.0)	0	0	22 (9.9)	0	0	14 (43.8)	0	0	2 (14.3)	0	0
Nausea	156 (35.1)	1 (0.2)	0	58 (26.1)	4 (1.8)	0	9 (28.1)	0	0	1 (7.1)	1 (7.1)	0
Alopecia	146 (32.9)	–	–	35 (15.8)	–	–	8 (25.0)	–	–	0	–	–
ALT increase	44 (9.9)	9 (2.0)	1 (0.2)	9 (4.1)	0	0	7 (21.9)	2 (6.3)	0	1 (7.1)	0	0
AST increase	43 (9.7)	11 (2.5)	0	11 (5.0)	2 (0.9)	0	6 (18.8)	1 (3.1)	0	1 (7.1)	0	0
Decreased appetite	66 (14.9)	3 (0.7)	0	20 (9.0)	0	0	6 (18.8)	1 (3.1)	0	0	0	0
Rash^a^	79 (17.8)	4 (0.9)	0	26 (11.7)	1 (0.5)	0	6 (18.8)	0	0	2 (14.3)	0	0
Arthralgia	148 (33.3)	3 (0.7)	–	75 (33.8)	1 (0.5)	–	5 (15.6)	0	–	3 (21.4)	0	–
Dry skin	55 (12.4)	0	–	13 (5.9)	0	–	5 (15.6)	0	–	0	0	–
Fatigue	166 (37.4)	8 (1.8)	0	61 (27.5)	1 (0.5)	0	5 (15.6)	0	0	1 (7.1)	0	0
Hot flush	93 (20.9)	0	–	68 (30.6)	0	–	2 (6.3)	0	–	4 (28.6)	0	–

– grade not available, *AE* adverse event, *ALT* alanine aminotransferase, *AST* aspartate aminotransferase, *LET* letrozole, *PAL* palbociclib, *PBO* placebo

^a^Clusters of preferred terms were used to represent multiple preferred terms

^b^One treatment-related death with lower respiratory tract infection and pulmonary embolism occurred in the PBO + LET group

The most common (> 20% incidence) nonhematologic AEs reported with palbociclib–letrozole in the Japanese patients were stomatitis (53.1% vs 30.4% in the overall population, respectively), nasopharyngitis (43.8% vs 14.0%), nausea (28.1% vs 35.1%), alopecia (25.0% vs 32.9%), and increased alanine aminotransferase (21.9% vs 9.9%) (Table 4). Stomatitis and nasopharyngitis were substantially (> 20% difference) more common among the Japanese vs overall population, whereas fatigue (15.6% vs 37.4%) was less common. Few patients had grade 3/4 nonhematologic AEs.

**Fig. 7:**
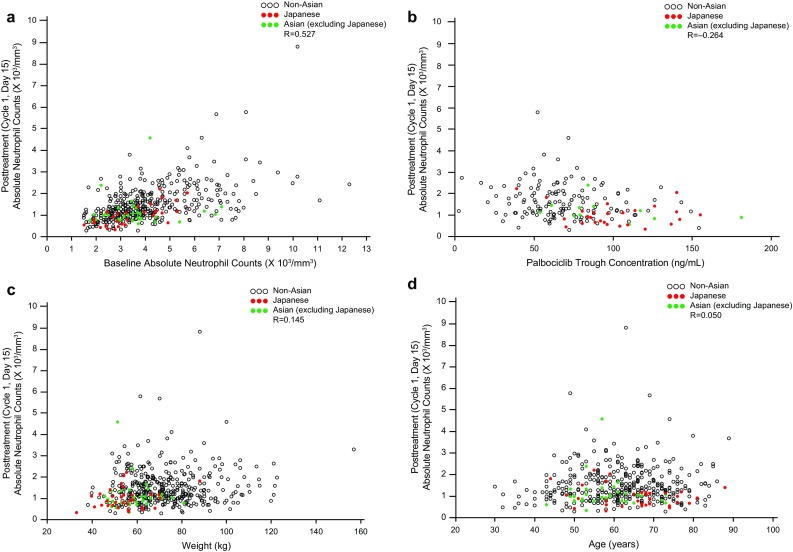
Posttreatment absolute neutrophil counts vs **a** baseline absolute neutrophil count, **b** palbociclib *C*_trough_, **c** body weight, and **d** age. Pearson product-moment correlation coefficients (*R*) are presented. Absolute neutrophil counts were assessed on day 15 of cycle 1. *C*_*trough*_ trough concentration

The overall incidence of AEs associated with dose reductions was higher in the Japanese vs overall population (62.5% vs 36.0%, respectively) (Table S2). Neutropenia (31.3%) and decreased neutrophil count (28.1%) were the only AEs associated with dose reductions in > 1 Japanese patient. Five Japanese patients permanently discontinued palbociclib because of AEs (neutropenia in 1 patient, neutrophil count decreased in 2 patients, cerebral hemorrhage and pulmonary fibrosis in 1 patient each). Posttreatment (Cycle 1, Day 15) absolute neutrophil counts (ANCs) correlated with baseline ANC in Japanese patients, other Asians (excluding Japanese), and non-Asians [overall correlation coefficient (*R*) = 0.527; Fig. 7a]. No apparent correlation was observed between posttreatment ANC and steady state *C*_trough_ (Fig. 7b), body weight (Fig. 7c), BSA/BMI (data not shown), or age (Fig. 7d).

## Discussion

This prespecified, exploratory subgroup analysis of PALOMA-2 suggests that palbociclib–letrozole is effective for postmenopausal Japanese women with ER+/HER2– ABC who have not received prior systemic treatment for ABC. The addition of palbociclib numerically increased PFS in Japanese patients, with a median investigator-assessed PFS of 22.2 vs 13.8 months with placebo–letrozole (HR, 0.59 [95%CI, 0.26–1.34]; 1-sided *P* = 0.1027). For other Asian patients (excluding Japanese) and for non-Asians, PFS was similar with palbociclib–letrozole (Fig. S1).

Based on the most recent data cutoff, median investigator-assessed PFS in Japanese patients was 24.9 months with palbociclib–letrozole vs 13.8 months with placebo–letrozole, while median PFS by BICR was 27.9 vs 16.6 months, respectively (Fig. 3). The difference between investigator- and BICR-assessed median PFS was also observed in the overall population (Figs. 1a, 2a), and was largely due to a higher censoring rate in the BICR analysis. Patients with investigator-diagnosed progressive disease were removed from study treatment and often switched to subsequent therapies with no further tumor assessment in the study. If progressive disease could not be confirmed on central review, the patient would be censored in the BICR analysis. Despite the difference in censoring rates, the results of BICR analysis showed preferable clinical benefit with palbociclib–letrozole and supported the findings of primary analysis.

Consistent with the overall population, ORR was higher in Japanese patients treated with palbociclib–letrozole; however, CBR was lower with palbociclib–letrozole than with placebo-letrozole. This discrepancy reflects differences in the number of Japanese patients who experienced disease progression within 24 weeks of randomization: 5 patients in the palbociclib–letrozole group vs 1 in the placebo–letrozole group.

In a single-arm phase 2 study of palbociclib–letrozole in Japanese women with treatment-naive ER+/HER2‒ ABC, the probability of PFS at 1 year was 75.0% (90%CI, 61.3%‒84.4%). Median PFS was not yet reached (lower limit of 95%CI was 21.7 months) and follow-up is ongoing [11].

In PALOMA-2, the geometric mean palbociclib *C*_trough_ at steady state was higher in Japanese (95.4 ng/mL) and other Asian patients (90.1 ng/mL) relative to non-Asians; however, individual values within each group were generally within range of one another. Although Japanese patients in the palbociclib–letrozole arm had a lower body weight relative to the overall population (median, 53.9 kg vs 68.0 kg), no apparent relationships were observed between *C*_trough_ and lower body weight/BSA/BMI. These results are consistent with those of a population PK analysis using data from other multinational studies of palbociclib, which showed that body weight had no clinically important effect on palbociclib PK and suggest there is no need for weight-based dosing [16].

Palbociclib is metabolized primarily by cytochrome P450 isozyme (CYP)3A and sulfotransferase (SULT) enzyme SULT2A1 [7]. There is wide interindividual variability in CYP3A metabolism, and analyses of cytochrome P450 activity among native Japanese, Chinese, Korean, and Caucasian populations indicate that CYP3A metabolism is independent of ethnicity and genotypes [17–19]. Although specific reasons were not identified for the observed difference in *C*_trough_ between the two populations in PALOMA-2, the interindividual variability of CYP3A might be a factor.

In the aforementioned open-label phase 2 study of palbociclib–letrozole in Japanese patients, full PK analysis in a palbociclib PK profile subset (*n* = 6) showed a remarkable similarity to that in non-Japanese patients enrolled in PALOMA-1 [11, 20]. In addition, in the global phase 3 study of palbociclib–fulvestrant (PALOMA-3), the within-patient mean steady state palbociclib *C*_trough_ in Japanese, Asian (excluding Japanese), and non-Asian patients demonstrated relative consistency in the central tendency and range of observed values across groups, indicating similar palbociclib exposure in these subpopulations [21]. Considered together, these data suggest there is no clinically relevant difference in PK between Japanese and non-Japanese patients.

Palbociclib–letrozole was well tolerated in Japanese patients, consistent with other palbociclib studies [6, 10, 11, 22, 23]; the most common AEs were neutropenia and leukopenia. A higher percentage of Japanese patients receiving palbociclib–letrozole experienced grade ≥ 3 neutropenia and grade ≥ 3 leukopenia compared with the overall population (87.5% vs 66.4% and 43.8% vs 24.8%, respectively); however, no febrile neutropenia was observed among Japanese patients.

Neutrophil counts vary among different ethnicities and ANC is generally lower in Asians vs non-Asians [24–26]. While historical comparisons must be interpreted cautiously, mean neutrophil counts reported for Japanese men (*n* = 3356) and women (*n* = 6027) were 3.8 (standard deviation, 1.3) $\times$ 10^3^/mm^3^ and 3.5 (1.3) $\times$ 10^3^/mm^3^, respectively [27], which are slightly lower than neutrophil counts previously observed in a non-Hispanic white population (*n* = 4270; 4.35 $\times$ 10^3^/mm^3^ [95%CI, 4.27−4.44]) [25]. In PALOMA-2, Japanese and most other Asians had baseline ANCs of < 6000/mm^3^, whereas many non-Asians had ANCs > 6000/mm^3^ (Fig. 7a). Lower baseline neutrophil counts in Japanese and other Asians could potentially explain the higher rate of neutropenia observed in these patients. Posttreatment ANC correlated with baseline neutrophil counts in all 3 groups (*R* = 0.527). Together, the data suggest that the higher incidence of neutropenia among Japanese patients was not related to a higher *C*_trough_ or lower body weight/BSA/BMI.

Although febrile neutropenia was not observed in Japanese patients in PALOMA-2, eight patients (1.8%, all non-Asian) in the overall population reported febrile neutropenia. Baseline neutrophil counts in these patients were relatively low: median 2430/mm^3^ (range, 1450–3300). These data suggest that lower baseline neutrophil counts may be a risk factor for febrile neutropenia as well as neutropenia with palbociclib, consistent with the widely reported association between lower baseline neutrophil counts and neutropenia/febrile neutropenia in patients receiving chemotherapy [28–30]. In vitro studies indicate that palbociclib causes reversible bone marrow suppression, clearly differentiating it from apoptotic cell death caused by cytotoxic chemotherapeutic agents [31]. This may explain the reduced frequency of febrile neutropenia seen with palbociclib vs cytotoxic chemotherapies.

Although common, neutropenia was effectively managed with dose modifications, and few Japanese patients permanently discontinued palbociclib because of this AE. In addition, the duration of PFS in Japanese patients was not affected by dose reduction to 100 or 75 mg QD (Fig. 4). Results from the open-label phase 2 study also suggest that dose reductions are unlikely to affect the efficacy of palbociclib in Japanese patients, although data from this study are immature and the impact of dose reductions on treatment response will require further evaluation [11]. Of note, dose reductions did not appear to compromise PFS in the overall populations of PALOMA-2 [32, 33] or PALOMA-3 [34, 35].

Stomatitis and nasopharyngitis were more commonly reported among Japanese patients receiving palbociclib–letrozole. All reported events in Japanese patients were grade 1 or 2, and no patients discontinued treatment or required dose reductions because of these AEs. The underlying cause for higher incidences of some AEs in Japanese patients is not clear. As described above, a lower body weight/BSA/BMI in Japanese patients does not necessitate dose adjustments, indicating that it is unlikely that these characteristics contributed to the higher incidence of certain AEs. Rather, differences in genetics, diet, or AE monitoring could possibly contribute to the observed differences.

This analysis suggests that the addition of palbociclib improved clinical outcomes in Japanese patients with ER+/HER2‒ ABC. However, these results should be interpreted cautiously, as the small sample size lacks the power to draw definitive conclusions, particularly regarding efficacy. The safety profile of palbociclib–letrozole was consistent with those reported previously. Hematologic toxicities were more common among Japanese patients than in the overall population, but were successfully managed with dose modifications. Taken together, the results seen in the Japanese and overall populations in PALOMA-2 suggest that palbociclib–letrozole merits consideration as a first-line treatment option for postmenopausal Japanese patients with ER+/HER2‒ ABC.

## Electronic supplementary material

Below is the link to the electronic supplementary material.

Supplementary material 1 (PDF 77 KB)

Supplementary material 2 (PDF 31 KB)

Supplementary material 3 (PDF 46 KB)
