# Supplementary material for: Palbociclib in combination with letrozole in patients with estrogen receptor–positive, human epidermal growth factor receptor 2–negative advanced breast cancer: PALOMA-2 subgroup analysis of Japanese patients
Source: Int J Clin Oncol. 2018 Dec 4;24(3):274–87. doi: 10.1007/s10147-018-1353-9 (PMC6399183; doi:10.1007/s10147-018-1353-9)
Supplement: Supplementary file 1 — Supplementary material 1 (PDF 77 KB) [file 10147_2018_1353_MOESM1_ESM.pdf]

Palbociclib in combination with letrozole in patients with estrogen receptor–positive, human epidermal growth factor receptor 2–negative advanced breast cancer: PALOMA-2 subgroup analysis of Japanese patients

Journal: International Journal of Clinical Oncology

Hirofumi Mukai,[a] Chikako Shimizu,[b] Norikazu Masuda,[c] Shoichiro Ohtani,[d] Shinji Ohno,[e] Masato Takahashi,[f] Yutaka Yamamoto,[g] Reiki Nishimura,[h] Nobuaki Sato,[i] Shozo Ohsumi,[j] Hiroji Iwata,[k] Yuko Mori,[l] Satoshi Hashigaki,[l] Yasuaki Muramatsu,[l] Takashi Nagasawa,[l] Yoshiko Umeyama,[l] Dongrui R. Lu,[m] Masakazu Toi[n]

Corresponding author:
Hirofumi Mukai, MD
Division of Breast and Medical Oncology
National Cancer Center Hospital East
6-5-1, Kashiwanoha
Kashiwa-shi, Chiba 277-8577, Japan
Ph: 04-7133-1111
Fax: 04-7131-4724
Email: hrmukai@east.ncc.go.jp

Fig. S1 Investigator-assessed PFS in non-Asian, Asian (excluding Japanese), and Japanese patients (ITT population)

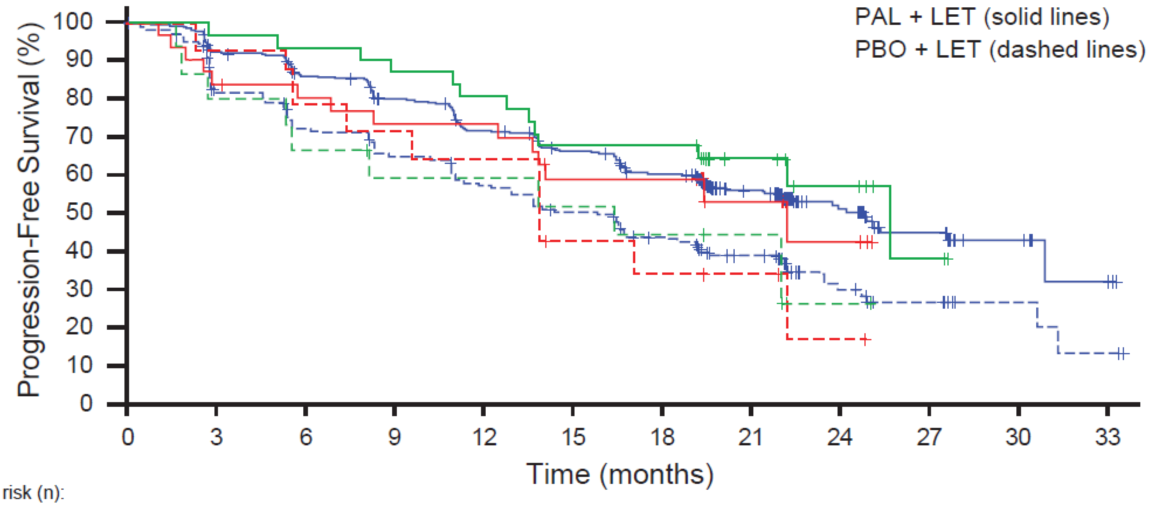

Patients at risk (n):

PAL + LET												
Japanese	32	26	23	21	21	16	16	8	4	0		
Asian (excluding Japanese)	33	30	29	27	25	21	21	12	7	2	0	
Non Asian	379	339	308	280	249	226	201	134	58	27	10	2
PBO + LET												
Japanese	14	13	11	10	9	5	4	3	1	0		
Asian (excluding Japanese)	16	12	10	8	8	7	6	5	1	0		
Non Asian	192	146	127	113	99	86	71	46	20	12	4	2

ITT intent-to-treat, *LET* letrozole, *PAL* palbociclib, *PBO* placebo, *PFS* progression-free survival
