# Supplementary material for: Palbociclib in combination with letrozole in patients with estrogen receptor–positive, human epidermal growth factor receptor 2–negative advanced breast cancer: PALOMA-2 subgroup analysis of Japanese patients
Source: Int J Clin Oncol. 2018 Dec 4;24(3):274–87. doi: 10.1007/s10147-018-1353-9 (PMC6399183; doi:10.1007/s10147-018-1353-9)
Supplement: Supplementary file 2 — Supplementary material 2 (PDF 31 KB) [file 10147_2018_1353_MOESM2_ESM.pdf]

Palbociclib in combination with letrozole in patients with estrogen receptor–positive, human epidermal growth factor receptor 2–negative advanced breast cancer: PALOMA-2 subgroup analysis of Japanese patients

Journal: International Journal of Clinical Oncology

Hirofumi Mukai,[a] Chikako Shimizu,[b] Norikazu Masuda,[c] Shoichiro Ohtani,[d] Shinji Ohno,[e] Masato Takahashi,[f] Yutaka Yamamoto,[g] Reiki Nishimura,[h] Nobuaki Sato,[i] Shozo Ohsumi,[j] Hiroji Iwata,[k] Yuko Mori,[l] Satoshi Hashigaki,[l] Yasuaki Muramatsu,[l] Takashi Nagasawa,[l] Yoshiko Umeyama,[l] Dongrui R. Lu,[m] Masakazu Toi[n]

Corresponding author:
Hirofumi Mukai, MD
Division of Breast and Medical Oncology
National Cancer Center Hospital East
6-5-1, Kashiwanoha
Kashiwa-shi, Chiba 277-8577, Japan
Ph: 04-7133-1111
Fax: 04-7131-4724
Email: hrmukai@east.ncc.go.jp

Table S1 Japanese sites and investigators who participated in the PALOMA-2 study

Site	Principal Investigator
Kyoto University Graduate School of Medicine	Masakazu Toi[a]
Aichi Cancer Center Hospital	Hiroji Iwata
National Cancer Center Hospital	Chikako Shimizu
National Cancer Center Hospital East	Hirofumi Mukai
National Hospital Organization Osaka National Hospital	Norikazu Masuda
Hakuaikai Medical Corporation Sagara Hospital	Yoshiaki Rai
Iwate Medical University Hospital	Masahiro Kashiwaba[b], Kazushige Ishida
National Hospital Organization Shikoku Cancer Center	Shozo Ohsumi
National Hospital Organization Kyushu Cancer Center	Shinji Ohno[b], Mayumi Ishida[b], Eriko Tokunaga
National Hospital Organization Hokkaido Cancer Center	Masato Takahashi
Chiba Cancer Center	Naohito Yamamoto
Hiroshima City Hiroshima Citizens Hospital	Kenji Higaki[b], Shoichiro Ohtani
Kumamoto University Hospital	Hirotaka Iwase
Saitama Cancer Center	Kenichi Inoue
Kumamoto City Hospital	Reiki Nishimura[b], Yasuhiro Okumura
Niigata Cancer Center Hospital	Nobuaki Sato

[a]Steering Committee member

[b]Previous Principal Investigator
