# Supplementary material for: Palbociclib in combination with letrozole in patients with estrogen receptor–positive, human epidermal growth factor receptor 2–negative advanced breast cancer: PALOMA-2 subgroup analysis of Japanese patients
Source: Int J Clin Oncol. 2018 Dec 4;24(3):274–87. doi: 10.1007/s10147-018-1353-9 (PMC6399183; doi:10.1007/s10147-018-1353-9)
Supplement: Supplementary file 3 — Supplementary material 3 (PDF 46 KB) [file 10147_2018_1353_MOESM3_ESM.pdf]

Palbociclib in combination with letrozole in patients with estrogen receptor–positive, human epidermal growth factor receptor 2–negative advanced breast cancer: PALOMA-2 subgroup analysis of Japanese patients

Journal: International Journal of Clinical Oncology

Hirofumi Mukai,[a] Chikako Shimizu,[b] Norikazu Masuda,[c] Shoichiro Ohtani,[d] Shinji Ohno,[e] Masato Takahashi,[f] Yutaka Yamamoto,[g] Reiki Nishimura,[h] Nobuaki Sato,[i] Shozo Ohsumi,[j] Hiroji Iwata,[k] Yuko Mori,[l] Satoshi Hashigaki,[l] Yasuaki Muramatsu,[l] Takashi Nagasawa,[l] Yoshiko Umeyama,[l] Dongrui R. Lu,[m] Masakazu Toi[n]

Corresponding author:
Hirofumi Mukai, MD
Division of Breast and Medical Oncology
National Cancer Center Hospital East
6-5-1, Kashiwanoha
Kashiwa-shi, Chiba 277-8577, Japan
Ph: 04-7133-1111
Fax: 04-7131-4724
Email: hrmukai@east.ncc.go.jp

Table S2 AEs associated with dose reduction in the overall population and Japanese patients (as-treated population)

	Overall Population		Japanese Patients	
**Adverse Event**	**PAL + LET (n=444)**	**PBO + LET (n=222)**	**PAL + LET (n=32)**	**PBO + LET (n=14)**
Any AE,[a] n (%)	160 (36.0)	3 (1.4)	20 (62.5)	1 (7.1)
Hematologic AEs				
Neutropenia	108 (24.3)	1 (0.5)	10 (31.3)	0
Neutrophil count decreased	23 (5.2)	0	9 (28.1)	0
Febrile neutropenia	6 (1.4)	0	0	0
Leukopenia	5 (1.1)	0	0	0
Nonhematologic AEs				
Asthenia	7 (1.6)	0	0	0
Fatigue	5 (1.1)	0	0	0
ECG QT prolonged	1 (0.2)	1 (0.5)	1 (3.1)	1 (7.1)
Cystitis	1 (0.2)	0	1 (3.1)	0
Pneumonia	1 (0.2)	0	1 (3.1)	0
Deep vein thrombosis	1 (0.2)	0	1 (3.1)	0

AE adverse event, *ECG* electrocardiogram, *LET* letrozole, *PAL* palbociclib, *PBO* placebo.

[a]AEs leading to dose reduction in ≥1% of patients in either arm of the overall population and in any Japanese patient.
